# Breaking away from an endemic state of multidrug-resistant *Pseudomonas aeruginosa* by daily sink disinfection

**DOI:** 10.1017/ash.2023.484

**Published:** 2023-11-15

**Authors:** Sumio Iwasaki, Rikako Sato, Keisuke Kagami, Kouji Akizawa, Kasumi Hayasaka, Tatsuya Fukumoto, Keisuke Taki, Yusuke Niinuma, Takehiro Yamada, Reiko Oyamada, Tsubasa Watanabe, Sho Nakakubo, Chiaki Watanabe, Takanori Teshima, Nobuhisa Ishiguro

**Affiliations:** 1 Department of Infection Control and Prevention, Hokkaido University Hospital, Sapporo, Hokkaido, Japan; 2 Division of Laboratory and Transfusion Medicine, Hokkaido University Hospital, Sapporo, Hokkaido, Japan; 3 Department of Pediatrics, Hokkaido University Graduate School of Medicine, Sapporo, Hokkaido, Japan; 4 Department of Pharmacy, Hokkaido University Hospital, Sapporo, Hokkaido, Japan; 5 Department of Pharmacotherapy, Hokkaido University of Science, Sapporo, Hokkaido, Japan; 6 Department of Respiratory Medicine, Faculty of Medicine, Hokkaido University, Sapporo, Hokkaido, Japan; 7 Department of Hematology, Hokkaido University Faculty of Medicine, Sapporo, Hokkaido, Japan

## Abstract

The detection rate of multidrug-resistant *Pseudomonas aeruginosa* in patients admitted to 2 wards and the intensive care unit decreased from 20.3% (129 of 636 isolates) to 4.2% (37 of 889 isolates) after the start of disinfection of hand washing sinks using alkyl diaminoethylglycine hydrochloride.

## Introduction

Multidrug-resistant *Pseudomonas aeruginosa* (MDRP) is associated with high mortality^1^ and outbreaks of MDRP have been reported.^2^ Here, we describe an endemic state due to MDRP for more than 5 years, the progress of measures, and the results of analysis.

## Background/setting

Our hospital is a 925-bed tertiary care hospital with 19 wards and an ICU. From 2002 to 2006, 8.9% to 42.3% of *Pseudomonas aeruginosa* (PA) isolates from patients in ward A (60 beds), ward B (60 beds), and the ICU (10 beds) were MDRP isolates (Supplementary Table 1). Isolation of MDRP carriers and education including the WHO’s five moments for hand hygiene using alcohol-based hand sanitizers were ineffective.

Considering the possibility that hand hygiene sinks (hereinafter referred to as sinks) had been reservoirs for MDRP, environmental cultures of sinks in 2 rooms each from ward A, ward B, and the ICU in which MDRP was detected from multiple patients were conducted. Before the start of disinfection, MDRP was detected in 3 of 6 sinks. Samples of bacterial culture were collected from 6 sinks at 1, 2, 4, 6, 8, 10, and 16 weeks after the introduction of disinfection with 1% solution of alkyl diaminoethylglycine hydrochloride (ADE) once every day. Multidrug-resistant *Pseudomonas aeruginosa* was detected in 1 of the 6 sinks one week after introduction but was not detected in the sinks after two weeks.

Disinfection of all sinks in ward A (42 sinks), ward B (44 sinks), and the ICU (38 sinks) with 1% solution of ADE once every day began in July 2007. The intervention using ADE is still ongoing. The detection rate of MDRP among PA isolates from patients in ward A, ward B, and the ICU after July 2007 was 4.2% (37 of 889 isolates), which was significantly lower than the detection rate of 20.3% (129 of 636 isolates) before June 2007 (*P* < 0.0001) (Table 1, Supplementary Table 2).

**Table 1.** Antimicrobial susceptibility and frequency of *P. aeruginosa* detected from ward A, ward B, and the ICU from May 2002 to June 2007 and from July 2007 to December 2017

Susceptibility			May 2002 to Jun. 2007	Jul. 2007 to Dec. 2017	*P* value (Fisher’s exact test)
IPM	AMK	LVFX			
R	R	R	129 (20.3%)	37 (4.2%)	<0.0001***
R	R	S	11 (1.7%)	14 (1.6%)	0.84
R	S	R	51 (8.0%)	71 (8.0%)	1
S	R	R	2 (0.3%)	7 (0.8%)	0.3192
R	S	S	83 (13.1%)	161 (18.1%)	0.0087**
S	R	S	14 (2.2%)	24 (2.7%)	0.6187
S	S	R	51 (8.0%)	64 (7.2%)	0.5565
S	S	S	295 (46.4%)	511 (57.5%)	<0.0001***
	(Total)		636 (100.0%)	889 (100.0%)	

Note. Statistically significant correlations given below: *P < .05, **P < .01, ***P < .001. IPM, imipenem; AMK, amikacin; LVFX, levofloxacin.

The detection rate of MDRP among PA isolates from patients in the nonintervention wards after July 2007 was 0.8% (13 of 1,623 isolates), which was significantly lower than the detection rate of 6.5% (73 of 1129 isolates) before June 2007 (*P* < 0.0001) despite the fact that disinfection of sinks was not implemented (Supplementary Table 3D).

This retrospective observation study was approved by the Review Board (IRB no. 017-0464).

## Methods for disinfection of hand hygiene sinks

(1) The sinks were rinsed with running water. (2) One percent solution of ADE was sprayed onto the sinks and left for 1 to 2 minutes. (3) The sinks were wiped with microfiber cloths to remove any dirt. (4) The sinks were rinsed with running water. (5) Procedure (2) was repeated. (6) The inside of each sink was wiped with a microfiber cloth. In ward A, ward B, and the ICU before the intervention by ADE, the sinks were cleaned with a sponge scrubber using neutral detergent and wiped with cloths. The sinks in nonintervention wards have been cleaned by the same procedure.

## Genotype analysis of MDRP isolates

Ninety-three of 166 MDRP isolates were used for genotyping. Sixty-nine MDRP isolates were identified as POT type 207-13 and sequence type 235 (ST 235) (Table 2, Supplementary Table 4). The MDRP isolates identified as POT types 207-13, 207-9, 558-57, and 558-61 had the *bla*_IMP_ gene detected by a PCR test included in the POT assay. The *bla*_IMP-1_ gene in 11 isolates of POT type 207-13 and 2 isolates of POT type 207-9 was confirmed by *bla*_IMP-1_ gene-specific PCR testing.

**Table 2.** Polymerase chain reaction (PCR)-based open reading frame (ORF) typing (POT) and multilocus sequence typing (MLST) of MDRP isolates detected from ward A, ward B, and the ICU

POT type (ST type)	2002	2003	2004	2005	2006	2007	2008	2009	2010	2011	2012	2013	2014	2015	2016	2017	Total
207-13 (235)			2	21	16	18	5	3		2	2						69
207-9 (235)				1								2	1		1		5
54-0 (170)				1							1						2
823-16 (274)									1	1							2
125-16 (2407)								1									1
153-0 (non-typable)									1								1
201-16 (829)								1									1
203-32 (308)					1												1
276-48 (217)							1										1
28-16 (186)							1										1
383-48 (244)																1	1
458-0 (1822)						1											1
558-57 (277)				1													1
558-61 (277)	1																1
573-56 (179)				1													1
575-16 (17)													1				1
575-20 (17)									1								1
636-16 (155)					1												1
823-40 (274)													1				1
NT (NT)	23	13	27	3	2	1	1				1	1	1				73
Number of MDRP isolates (%)	24 (23.8)	13 (10.6)	29 (26.6)	28 (22.4)	20 (15.3)	20 (19.8)	8 (6.5)	5 (4.9)	3 (3.5)	3 (3.0)	4 (4.9)	3 (3.4)	4 (5.1)	0 (0.0)	1 (1.8)	1 (1.9)	166 (10.9)
Number of *P. aeruginosa* isolates	101	123	109	125	131	101	124	102	85	99	82	89	79	68	55	52	1525

### Correlations between antibiotic use and susceptibility to P. aeruginosa

The amount of carbapenem used had a negative correlation with the number of MDRP isolates (ρ = −0.8393; *P* < 0.0001) and a positive correlation with the number of PA isolates that were susceptible to all carbapenems, aminoglycosides, and quinolones (ρ = 0.7643; *P* = 0.0009) (Supplementary Tables 5, 6). The amount of aminoglycoside used had a positive correlation with the number of MDRP isolates (ρ = 0.6607; *P* = 0.0073) and a negative correlation with the number of PA isolates that were susceptible to all carbapenems, aminoglycosides, and quinolones (ρ = −0.75; *P* = 0.0013).

## Discussion

In this report, we described an endemic state of several MDRP strains. Seventy-four (79.6%) of 93 MDRP isolates tested for genotype were identified as sequence type 235 (ST 235) and had the *bla*_IMP_ gene (Table 2). ST235 and their close variants have been shown to be prominent in MDRP strains that carry the gene encoding metallo-β-lactamase.^3,4^

A considerable number of studies have provided evidence that sink drainage systems are reservoirs for hospital-acquired infection due to gram-negative bacteria such as PA.^5^ Daily disinfection of sinks does not eliminate PA in the water pipes under the sinks, but it can at least temporarily remove PA in the sinks and reduce the transmission of MDRP to the hands.

One percent ADE was used as a disinfectant to resolve the endemic state of MDRP described here. *Pseudomonas aeruginosa* has been reported to be completely killed by exposure to 0.2% ADE for 0.5 min.^6^ If daily disinfection of sinks becomes widespread, reduced susceptibility of bacteria to disinfectants could become a major problem, and surveillance of the susceptibility of bacteria to disinfectants is a future issue.

Restriction of carbapenem usage as part of a multifaceted infection control strategy against MDRP has been reported.^7^ In the present study, MDRP reduction was achieved without limiting the use of carbapenems. The positive correlation between the use of carbapenems and the number of PA isolates that were susceptible to carbapenems, aminoglycosides, and quinolones disappeared when MDRP was excluded from the analysis (data not shown). Decrease in the use of aminoglycosides is a nationwide trend in Japan^8^ and was not considered to be directly related to the decrease in the number of MDRP isolates (Supplementary Tables 5, 6).

Our study has several limitations. (1) The MDRP strains isolated from sinks were not stored and whole-genome sequencing of MDRP strains was not performed. (2) It is necessary to verify whether daily disinfection of sinks affects colonization of PA in the drainpipes. (3) In addition to sinks, wards have wet environments such as slop sinks. It remains to be determined how far the disinfection should be expanded. (4) The movement of MDRP carriers between wards, adherence to isolation precautions, compliance with hand hygiene using alcohol-based hand sanitizers, testing of MDRP carriers, adherence to hand hygiene, and antimicrobial use should be investigated. These are key factors for detecting why MDRP detection rate declined in nonintervention wards.

## Conclusions

Daily disinfection of sinks using ADE helped to break out of an endemic state of MDRP. This method is worth trying in endemic states or outbreaks of MDRP.

## Supporting information

Iwasaki et al. supplementary material Iwasaki et al. supplementary material

## Supplementary material

For supplementary material accompanying this paper visit http://doi.org/10.1017/ash.2023.484. click here to view supplementary material
