# Supplementary material for: Breaking away from an endemic state of multidrug-resistant Pseudomonas aeruginosa by daily sink disinfection
Source: Antimicrob Steward Healthc Epidemiol. 2023 Nov 15;3(1):e209. doi: 10.1017/ash.2023.484 (PMC10753494; doi:10.1017/ash.2023.484)
Supplement: Iwasaki et al. supplementary material [file S2732494X23004849sup001.pdf]

Materials and Methods

Environmental culture of hand washing sinks

The procedure for sampling and culturing of the sinks was as follows: The bowl and drain of each sink were wiped with sterile gauze. The gauze was soaked in 50 mL of sterile water in a Falcon tube for 15 minutes. After the gauze had been removed, the Falcon tube was centrifuged at 3500 rpm for 15 minutes. An aliquot of the pellet was applied to blood agar and DHL media, and bacteria that grew after 48 hours were identified.

Bacterial identification and susceptibility testing

Identification and antimicrobial susceptibility tests for the bacterial isolates were carried out using the Microscan WalkAway 96 Plus system (Beckman Coulter, CA, USA). In Japan, MDRP is defined as *P. aeruginosa* that is resistant to carbapenems, aminoglycosides and fluoroquinolones. Minimum inhibitory concentration (MIC) breakpoints for imipenem, amikacin, ciprofloxacin and levofloxacin are ≥16, ≥32, ≥4 and ≥8 mg/mL, respectively (Antibiotics (Basel). 2021 Sep 30;10(10):1189. doi: 10.3390/antibiotics10101189.).

P. aeruginosa isolates obtained from patients between May 2002 and December 2017 were divided into eight groups according to their antimicrobial

susceptibility patterns: (1) IPM (R), AMK (R) and LVFX (R), (2) IPM (R), AMK (R) and LVFX (S), (3) IPM (R), AMK (S) and LVFX (R), (4) IPM (S), AMK (R) and LVFX (R), (5) IPM (R), AMK (S) and LVFX (S), (6) IPM (S), AMK (R) and LVFX (S), (7) IPM (S), AMK (S) and LVFX (R), (8) IPM (S), AMK (S) and LVFX (S). Next, for each group of *P. aeruginosa*, only isolates that were first detected in patients were extracted. For example, if a patient was initially admitted to ward A and then transferred to the ICU, and MDRP was detected in both ward A and the ICU, only the MDRP detected from ward A was counted. If multiple strains of *P. aeruginosa* with different antimicrobial susceptibility patterns were detected in a patient, each strain was included in the number of strains in wards or the ICU in which it was first detected.

Genotype analysis

Genotype analysis was performed on MDRP isolates by the polymerase chain reaction (PCR)-based open reading frame typing (POT) system using a Cica Geneus Pseudo POT Kit (Kanto Chemical, Tokyo, Japan) as previously described (J Appl Microbiol. 2016 Feb;120(2):487-97. doi: 10.1111/jam.13016.). The Cica Geneus Pseudo POT Kit contains primer pairs designed to detect the metallo-β-lactamases (MBLs) bla_{IMP} and bla_{VIM} (J Appl Microbiol. 2016

Feb;120(2):487-97. doi: 10.1111/jam.13016.). Multi locus sequence typing (MLST) was performed on MDRP isolates by using the primers specified in the MLST website (https://pubmlst.org/index.php/organisms/pseudomonas-aeruginosa), and the sequence types (STs) were assigned through the same P. aeruginosa MLST database (Wellcome Open Res. 2018 Sep 24;3:124. doi: 10.12688/wellcomeopenres.14826.1.).

Detection of the $bla_{\mathrm{IMP\text{-}1}}$ gene was performed by PCR (J Hosp Infect. 2011 Aug;78(4):317-22. doi: 10.1016/j.jhin.2011.04.013.)

Antibiotic use

Information on the amounts of antibiotics (carbapenems, aminoglycosides and quinolones) used in wards and the ICU from July 2003 to December 2017 was obtained from the database system of the hospital. Antibiotic use was evaluated on the basis of the antibiotic use density (AUD), which was defined as antibiotic use (in grams)/defined daily dose (DDD) per 100 patient-days. DDD is the assumed average maintenance dose per day for a drug used for its main indication in adults, as assigned by the World Health Organization (WHO).

Statistical analysis

Trends in detection of resistant bacteria and trends in antibiotic use were analyzed by Pearson's correlation test. Comparisons of the rates of detection of resistant bacteria before and after disinfection were analyzed by Fisher's exact test. Correlations between detection of resistant bacteria and antibiotic use were analyzed by Spearman's correlation tests. For all comparisons, P values < 0.05 were considered statistically significant. The calculations were performed using JMP Pro version 14 (SAS Institute Inc., Japan).
